# Multimodal neuroimmune diagnostic profiling for pediatric lower urinary tract dysfunction associated with occult sacral nerve abnormalities

**DOI:** 10.3389/fimmu.2026.1947984

**Published:** 2026-10-06

**Authors:** Dan Wang, Jun Pei, Hongyong Liao, Yimeng Liu, Yuangui Yang, Min Deng, Nini An

**Affiliations:** 1 Department of Pediatric Surgery, Guizhou Provincial People’s Hospital, Guiyang, China; 2 Department of Pediatric Urology, Shanghai Children’s Medical Center GuiZhou Hospital, Shanghai Jiao Tong University School of Medicine, Guiyang, China; 3 Department of Children’s Ultrasound, Shanghai Children’s Medical Center GuiZhou Hospital, Shanghai Jiao Tong University School of Medicine, Guiyang, China

**Keywords:** bladder remodeling, diagnostic prediction model, mucosal immunity, neuroimmune signaling, occult sacral neuropathy, pediatric lower urinary tract dysfunction, urinary inflammatory biomarkers

## Abstract

**Background:**

Persistent pediatric lower urinary tract dysfunction (LUTD) may be associated with occult sacral neural impairment, a clinically challenging condition characterized by subtle sacral pathway dysfunction without overt spinal dysraphism.

**Objective:**

To develop, internally validate, and externally validate a multimodal diagnostic model integrating ultrasound, urodynamics, neuroelectrophysiology, and urinary biomarkers.

**Methods:**

The development dataset comprised 120 records (45 occult sacral neuropathy-related LUTD, 45 functional non-neurogenic LUTD, and 30 controls). The target-condition label followed a prespecified multidisciplinary composite reference standard based on clinical phenotype, neurological examination, spinal/pelvic MRI when available, conventional urodynamic interpretation, and longitudinal course; biomarker values and model probabilities were not components of that label. Preanalytical metadata included pre-catheterization collection, centrifugation at 3000 or 5000 x g for 10 minutes, 1.0- 1.5 mL aliquots, storage at -80 °C, limited freeze-thaw exposure, and creatinine normalization. We internally validated a six-predictor logistic model with 1,000 bootstrap repetitions. We assessed a separate 90-record, two-site dataset without refitting or threshold optimization.

**Results:**

The locked model had an apparent AUC of 0.937 and Brier score of 0.099; optimism-corrected values were 0.920 and 0.116, respectively. The bootstrap mean calibration intercept was -0.065, and the slope was 1.032. In the external transportability analysis, the AUC was 0.928 (95% CI, 0.861-0.977), the Brier score was 0.098, the calibration intercept was -0.400, the calibration slope was 0.887, sensitivity was 80.0%, and specificity was 88.3%.

**Conclusion:**

Multimodal neuroimmune profiling demonstrated strong discrimination and acceptable calibration after optimism correction and external validation in an independent two-site cohort. Prospective multicenter validation is required before clinical implementation.

## Graphical Abstract

**Figure:**
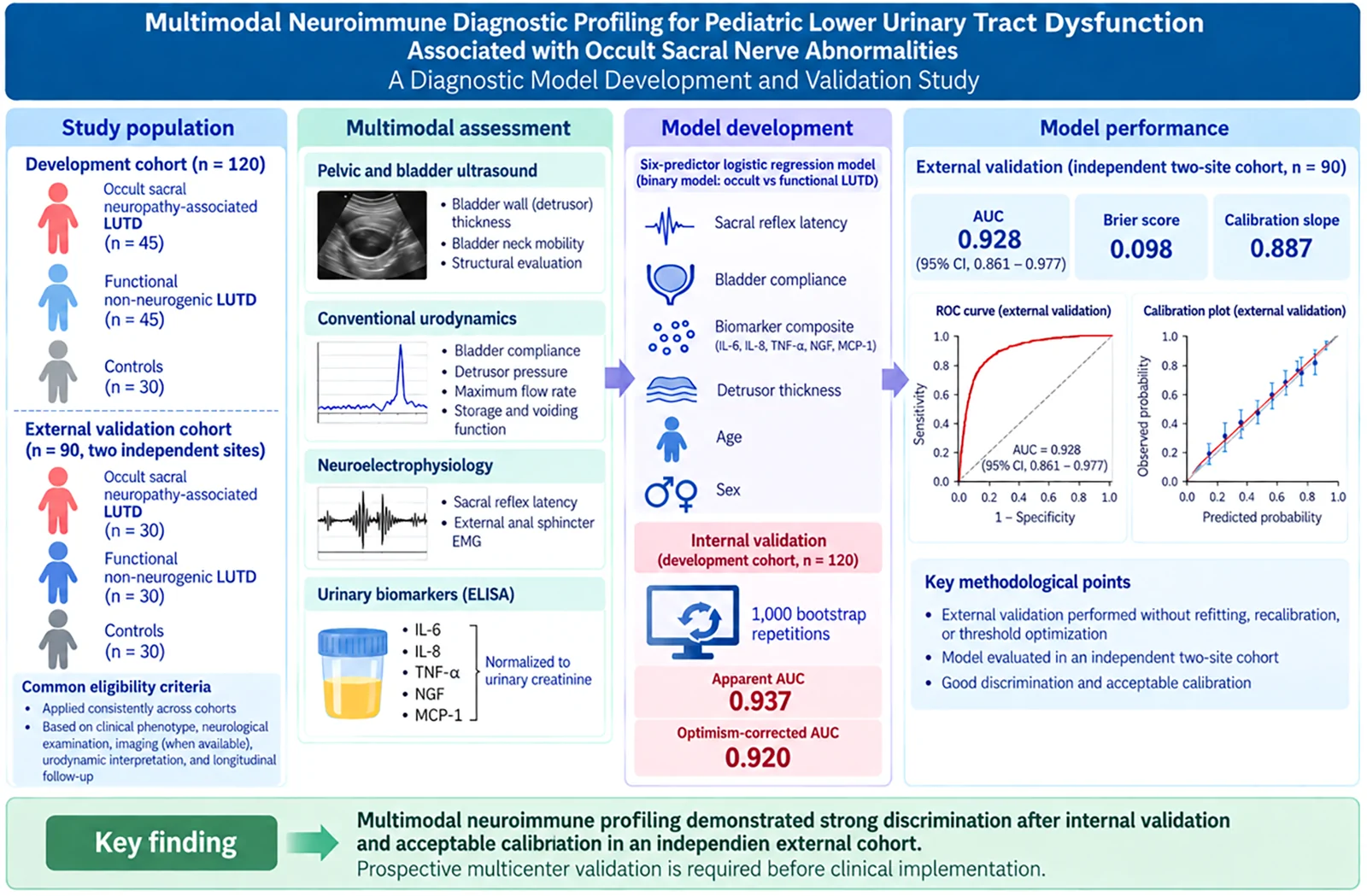

## 1 Introduction

Pediatric lower urinary tract dysfunction (LUTD) is a clinically significant continuum of storage and voiding urinary dysfunction that can lead to urinary incontinence, frequent urinary tract infection, residual urine, bladder remodeling, psychosocial stress, and possible upper urinary tract trauma in cases of high-pressure bladder dysfunction. Pediatric nomenclature is crucial because clinical terminology changes with age, continence maturation, bowel processes, and neurodevelopment; therefore, adult nomenclature does not apply directly to children (1, 2). Neurogenic lower urinary tract dysfunction (NLUTD) has been historically accepted concerning children with evident spinal dysraphism, myelomeningocele, or spinal injury, or known neurological disease; however, a challenging diagnostic group comprises children with suspected occult sacral nerve abnormalities but a persistent LUTD despite the absence of overt external spinal abnormality or initial definite radiographic evidence (3, 4).

In clinical practice, these children can first be defined as having functional non-neurogenicvoiding dysfunction because the urgency, frequency, continuity of the urinary stream, daytime wetting, straining, and post-void urine residue largely overlap between neurogenic and non-neurogenic phenotypes. Traditional assessment is based on history, voiding diary, physical examination, urinalysis, renal-bladder ultrasound, uroflowmetry, and the chosen urodynamic evaluation. These approaches are useful, though they often characterize the final bladder phenotype rather than the upstream neural pathway. Urodynamic studies remain a significant method for assessing pediatric LUTD. However, interpretation has been reported to require child-specific methods, artifact control, age-adjusted estimates of bladder capacity, and standardized reporting (2). Imaging urodynamics can describe detrusor compliance, flow patterns, and coordination of sphincter activity, but not specifically sacral neural impairment or immune-mediated tissue maintenance (**Supplementary Table S1**).

High-frequency perineal or rectal ultrasound provides an additional dynamic structural assessment of bladder neck mobility, detrusor morphology, urethral sphincter thickness, pelvic floor behavior, and post-void structural changes. Neuroelectrophysiology, such as pelvic floor electromyography (EMG) and sacral reflex latency, provides functional evidence of neuromuscular coordination and sacral pathway integrity. Nevertheless, none of the modalities is likely to identify any individual among occult sacral neuropathy, severe functional voiding dysfunction, and transient urinary symptoms in any reliable way. This limitation supports a multimodal diagnostic approach that combines anatomic imaging, functional pressure-flow data, electrophysiological findings, and biological signatures (**Supplementary Table S1**).

The neuroimmune framework is scientifically pertinent, as bladder management is not purely mechanical. The urothelium is a stretch-receptive, immunologically active interface that reacts to stretch, neural factors, infection-related stimuli, and inflammatory mediators. Experimental and translational evidence shows that lower urinary tract function relies on coordinated communication among urothelial cells, afferent nerves, autonomic efferent nerves, hiatal smooth muscle, and immune mediators (5–7). Nowadays, neuroimmune communication is widely accepted as a general biological process in which nervous system activity can influence immune activation and tissue homeostasis (8). Chronic neural dysregulation in the bladder can promote sustained urothelial stress, disrupted afferent signaling, cytokine release, mast cell or macrophage stimulation, and progressive detrusor remodeling. In neurogenic and non-neurogenic LUTD, overactive bladder, interstitial cystitis/bladder pain syndrome, and bladder sensory disorders, urinary biomarkers have been examined, including interleukin-6 (IL-6), interleukin-8 (IL-8), tumor necrosis factor-alpha (TNF-alpha), nerve growth factor (NGF), and monocyte chemoattractant protein-1 (MCP-1). Still, pediatric evidence is limited and heterogeneous (9–12). **Figure 1** illustrates the proposed biological framework linking occult sacral neural dysfunction, autonomic dysregulation, urothelial immune activation, inflammatory signaling, and bladder remodeling.

**Figure 1:**
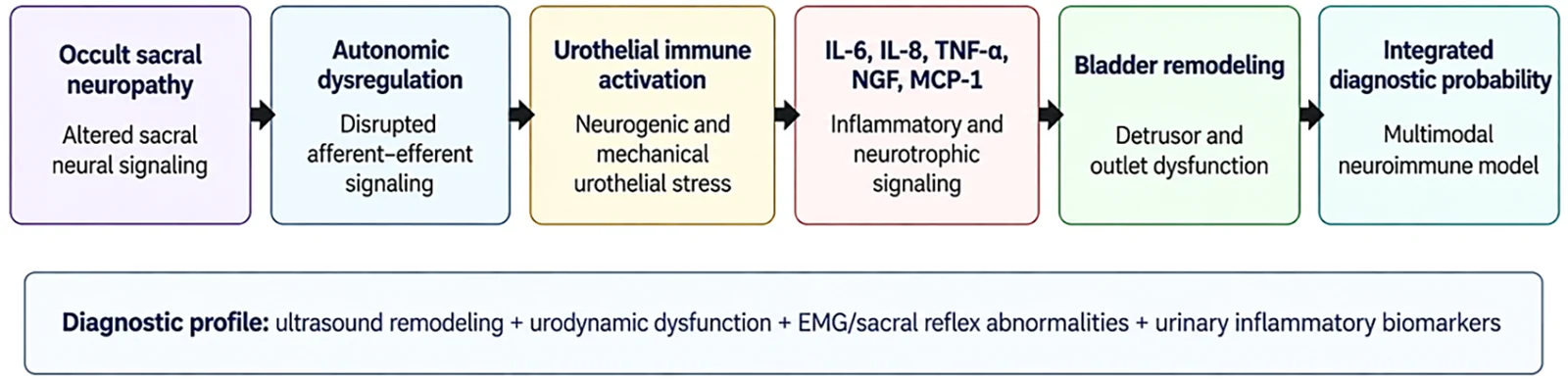
Neuroimmune diagnostic profiling in pediatric occult sacral neuropathy-associated LUTD.

The key gap addressed was the lack of a clear pediatric model linking occult sacral neural dysfunction, bladder remodeling, urodynamic impairment, neuroelectrophysiological abnormalities, and urinary inflammatory activation. We derived development and external validation records from institutional clinical data and archived urine specimens to reflect clinically plausible distributions and associations. We hypothesized that children with occult sacral nerve abnormalities would exhibit a characteristic neuroimmune LUTD phenotype with neural dysregulation, increased inflammatory biomarkers, and functional bladder remodeling, and that integrated multimodal profiling would capture this more effectively than any single diagnostic domain (7–9). The analyses evaluated model development, internal validation, and external validation (transportability).

Considering the current literature, multiple urinary biomarker investigations have compared inflammatory or neurotrophic biomarkers in adult samples from patients with bladder pain, overactive bladder, or mixed LUTD (12–15). Still, comparatively few have compared biomarker signals with pediatric neurophysiology and structural bladder outcome measures. A model using only Biomarkers would be biologically incomplete, since elevated cytokines can indicate infection, irritation, bladder overdistension, or nonspecific inflammation. Conversely, a purely urodynamic model can overlook low-grade neural trauma and immune stimulation that precede observable pressure-flow degradation. The current research thus viewed biomarkers as a single component of a broader neuroimmune phenotype, rather than as independent diagnostic tests (7–9).

### 1.1 Objectives and study hypotheses

#### 1.1.1 Primary objective

To assess the diagnostic validity of a combined model comprising high-frequency perineal/rectal ultrasound, urodynamic variables, neuroelectrophysiological variables, and urinary inflammatory biomarkers to distinguish occult sacral neuropathy-related LUTD from functional non-neurogenic LUTD and age-matched controls. The main outcome was the area under the receiver operating characteristic curve (AUC) of the integrated model.

#### 1.1.2 Secondary objectives

To determine relationships among cytokine concentrations, bladder structural remodeling, urodynamic dysfunction, electrophysiological dysfunction, and symptom severity; compare biomarker expression across diagnostic groups; and develop an exploratory neuroimmune risk model for occult sacral neuropathy-related bladder dysfunction. The working mechanistic model identified the following cascade: occult sacral neuropathy appeared to cause autonomic dysregulation, urothelial immune activation, cytokine-mediated remodeling, and clinically relevant voiding dysfunction.

#### 1.1.3 Study hypothesis

Children with occult sacral nerve abnormalities would exhibit a characteristic neuroimmune LUTD phenotype compared with children with functional non-neurogenic LUTD or transient urinary symptoms.

## 2 Methodology

### 2.1 Study design and cohort structure

We used a retrospective pediatric diagnostic-model cohort design (**Figure 2**). The development dataset included 120 complete records: 45 occult sacral neuropathy-associated LUTD, 45 functional non-neurogenic LUTD, and 30 controls obtained from Shanghai Children’s Medical Center Guizhou Hospital. An independent external validation dataset comprised 90 records from two sites at Guizhou Provincial People’s Hospital, including 30 children with occult sacral neuropathy-associated LUTD, 30 with functional non-neurogenic LUTD, and 30 controls, which was independent of the development cohort and used the same prespecified eligibility criteria, predictor definitions, and reference-standard adjudication framework. We applied the locked six-predictor model to the external dataset using the development-cohort centering and scaling constants, without refitting coefficients, reselecting predictors, recalibrating, or optimizing thresholds. Predictor definitions were the same across datasets, while site-specific distributions differed, allowing us to evaluate transportability. We collected medical data and records between January 2020 and December 2025.

**Figure 2:**
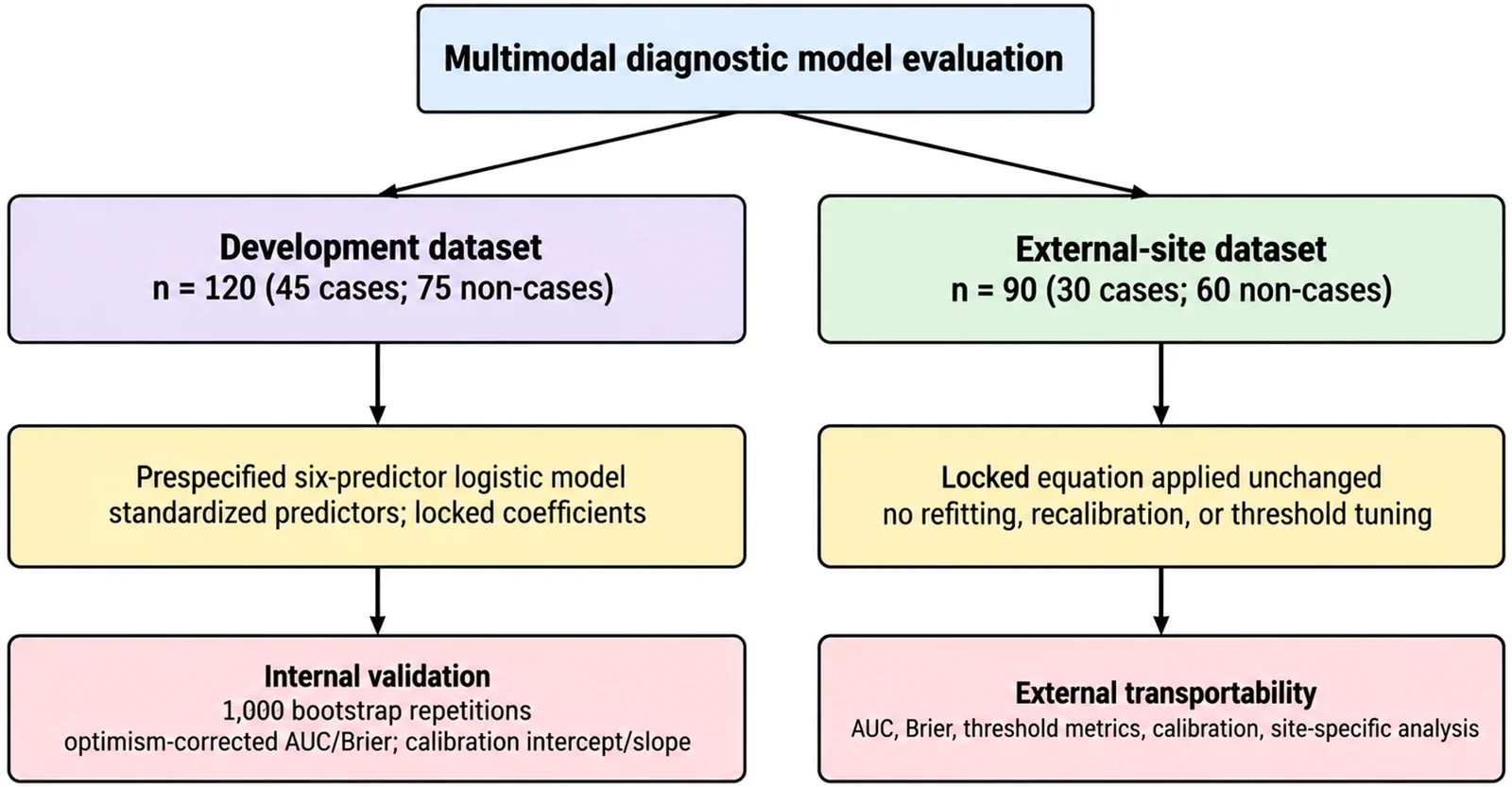
Model development, bootstrap validation, and external transportability workflow.

### 2.2 Eligibility

Exclusion criteria were overt congenital spinal dysraphism, spina bifida or myelomeningocele, active urinary tract infection, prior spinal or pelvic surgery, major congenital malformation of the urinary tract, missing locked predictors, or incomplete biomarker records. Multidisciplinary clinical consensus, based on clinical manifestations, spinal or pelvic MRI findings where present, neurological examination, urodynamic interpretation, and longitudinal follow-up, served as the basis for establishing the diagnostic reference standard. We processed the external validation dataset in the same way.

We used electronic medical records, structured urology assessment forms, voiding diaries, parent questionnaires, and archived diagnostic reports to derive clinical variables. Imaging variables comprised bladder neck mobility, detrusor morphology, bladder wall or detrusor appearance, urethral sphincter thickness, and other pertinent post-void findings of high-frequency perineal or rectal ultrasound. Urodynamic variables included bladder compliance, detrusor pressure during filling and voiding, maximum flow rate, pressure-flow pattern, residual urine, and detrusor-sphincter dyssynergy or sphincter non-relaxation. Neuroelectrophysiological outcomes were pelvic floor EMG activity and sacral reflex latency. We used standardized data extraction forms to review all available imaging and functional reports and minimize inter-reviewer differences.

### 2.3 Reference standard for occult sacral neuropathy

There is no definite test for occult sacral neuropathy. Two independent reviewers from the Pediatric Department of Shanghai Children’s Medical Center, GuiZhou Hospital, adjudicated the reference-standard diagnosis. Reviewers assessed the prespecified clinical, neurological, imaging, urodynamic, and longitudinal criteria without access to the biomarker composite or model probabilities. Disagreements were resolved by consensus. We applied the same adjudication framework to the development and external validation cohorts. The target-condition label followed a prespecified hierarchical composite reference criterion in line with diagnostic-accuracy precepts (16). Its components were persistent clinical phenotype, standardized neurological findings, spinal or pelvic MRI when clinically available, conventional urodynamic interpretation, and longitudinal clinical course (**Supplementary Table S2**). MRI was supportive when imaging was equivocal, and no single urodynamic abnormality was determinative. The biomarker composite and integrated model probability were not included in the reference-standard label, thereby preventing direct incorporation of the model output into its target. We defined the persistent LUTD phenotype based on longitudinal persistence of lower urinary tract symptoms and the prespecified reference-standard criteria rather than on treatment response. The retrospective dataset did not systematically capture detailed prior bladder-directed medication exposure, so we did not incorporate it into phenotype classification.

### 2.4 Index tests and data extraction

We created variables and verified them against a prespecified data dictionary based on the manuscript framework and published research on pediatric lower urinary tract. Ultrasound variables included bladder-neck movement, detrusor thickness, urethral-sphincter thickness, and post-void structural results. Urodynamic variables included bladder compliance, detrusor pressure, maximum flow rate, residual urine, and outlet discoordination. Pelvic-floor EMG activity and sacral-reflex latency were neuroelectrophysiological variables. Before model analysis, we checked range, coding, completeness, and cross-variable consistency.

### 2.5 Urinary biomarker handling and storage metadata

To address preanalytical variation, we recorded explicit biomarker-handling metadata for each case. As shown in **Supplementary Table S3**, the protocol specified urine collection before urodynamic catheterization, centrifugation at 3000 or 5000 x g for 10 minutes, aliquot volumes of 1.0 or 1.5 mL, uninterrupted storage at -80°C, and no more than two freeze-thaw cycles. Storage duration had a median of 33.5 months (IQR, 18.0-41.3; range, 1-60) in development and 25.5 months (IQR, 15.3-36.0; range, 1-47) in the external dataset. We quantified urinary IL-6, IL-8, TNF-α, NGF, and MCP-1 using commercially available human ELISA kits (Elabscience Biotechnology Inc., Wuhan, Hubei, China) according to the manufacturer’s instructions. We analyzed samples in duplicate and determined concentrations from plate-specific standard curves with appropriate assay controls. Urine samples were centrifuged at 3000–5000 × *g* for 10 min, aliquoted into 1.0 to 1.5 mL volumes, stored continuously at −80°C, and subjected to no more than two freeze–thaw cycles. Laboratory personnel were blinded to diagnostic classification. Biomarker concentrations were normalized to urinary creatinine and expressed as pg/mg creatinine. These parameters were consistent with published urine-biomarker processing studies and pediatric urinary NGF studies (17, 18).

### 2.6 Model development and internal validation

The composite-reference-standard diagnosis of occult sacral neuropathy-related LUTD was a binary result. We based the prespecified predictors on six domains: sacral-reflex latency, bladder compliance, biomarker composite score, detrusor thickness, age, and sex (**Supplementary Table S4**).

We retained continuously measured predictors on their original scale and standardized them using development-cohort means and population standard deviations. We defined the biomarker composite as the average of standardized natural-log IL-6, IL-8, TNF-alpha, NGF, and MCP-1 concentrations.

We built and locked a multivariable logistic model; we validated it internally using 1000 bootstrap repetitions and evaluated performance. We then calculated optimism as bootstrap apparent performance minus bootstrap test performance, and compared it to the original apparent performance. We quantified discrimination using AUC and overall prediction error using a Brier score. We evaluated calibration intercept with a logistic model in which the outcome was regressed on the predicted logit as an offset; we evaluated calibration slope by regressing the logit outcome on the predicted logit using the freely estimated intercept and slope. Reporting followed the Transparent Reporting of a Multivariable Prediction Model for Individual Prognosis or Diagnosis plus Artificial Intelligence (TRIPOD+AI) statement and checklist published by Collins et al. (19), which provides guidance for transparent reporting of prediction-model development and validation studies (19).

### 2.7 External validation (transportability) analysis

We applied the locked equation to the 90-record external-site dataset, using the development-cohort centering and scaling constants and a threshold of 0.50 without modification (**Supplementary Table S5**). We did not refit coefficients, recalibrate, select predictors, or optimize thresholds. We evaluated pooled and site-specific AUCs, Brier score, sensitivity, specificity, positive and negative predictive values, calibration intercept, and calibration slope. We used stratified nonparametric bootstrap resampling to obtain 95 percent confidence intervals. This analysis evaluated transportability in the independent external dataset.

Secondary analyses included group biomarker distributions and correlations between cytokines, ultrasound remodeling, urodynamics, EMG, sacral-reflex latency, and symptom severity. We chose Pearson or Spearman correlation based on distributional considerations. We included age and sex in the locked equation to account for developmental and demographic variability. All locked-model predictors were complete in both the development and validation datasets; therefore, we did not impute data for the primary analysis.

We checked and removed identifiers before analysis; compared ranges and units with the data dictionary; corrected logically impossible combinations; and confirmed completeness checks showed all locked predictors had complete data. We compared distributional summaries across the development and external validation cohorts to identify differences in case mix and predictor distributions. Biomarker quality-control checks included standard-curve, duplicate-well, and batch-effect verification.

We mitigated potential bias through prespecified predictor definitions, separating model probabilities from label generation, bootstrap validation, and locked external application. Reporting followed STARD and TRIPOD+AI guidance on reference-standard description, model specification, discrimination, calibration, and interpretation (16, 19).

Statistical analyses were performed using IBM SPSS Statistics version 29.0 (IBM Corp., Armonk, NY, USA) and R version 4.4.1 (R Foundation for Statistical Computing, Vienna, Austria). Analyses included multivariable logistic regression, 1,000-repetition bootstrap internal validation, ROC/AUC analysis, Brier score estimation, calibration intercept and slope assessment, Pearson or Spearman correlation analysis, and exploratory random-forest feature-importance analysis. The random-forest analysis was exploratory and was not used for predictor selection or development of the primary diagnostic model. All statistical tests were two-sided, with P < 0.05 considered statistically significant.

### 2.8 Ethics statement

The retrospective study was conducted in accordance with the Declaration of Helsinki. It was approved by the Medical Ethics Committee of Shanghai Children’s Medical Center Guizhou Hospital, Shanghai Jiao Tong University School of Medicine (Approval No. 2026 (XJS) -012). We waived informed consent because we used retrospective anonymous records. In accordance with the ethics approval, we analyzed all data anonymously and reported only aggregate data.

## 3 Results

### 3.1 Cohort assembly and diagnostic grouping

The development dataset consisted of 120 complete cases: 45 target-condition cases, 45 functional non-neurogenic LUTD comparators, and 30 controls. The external transportability dataset included 90 complete observations from two sites: 30 target-condition cases and 60 non-cases (**Figure 3**).

**Figure 3:**
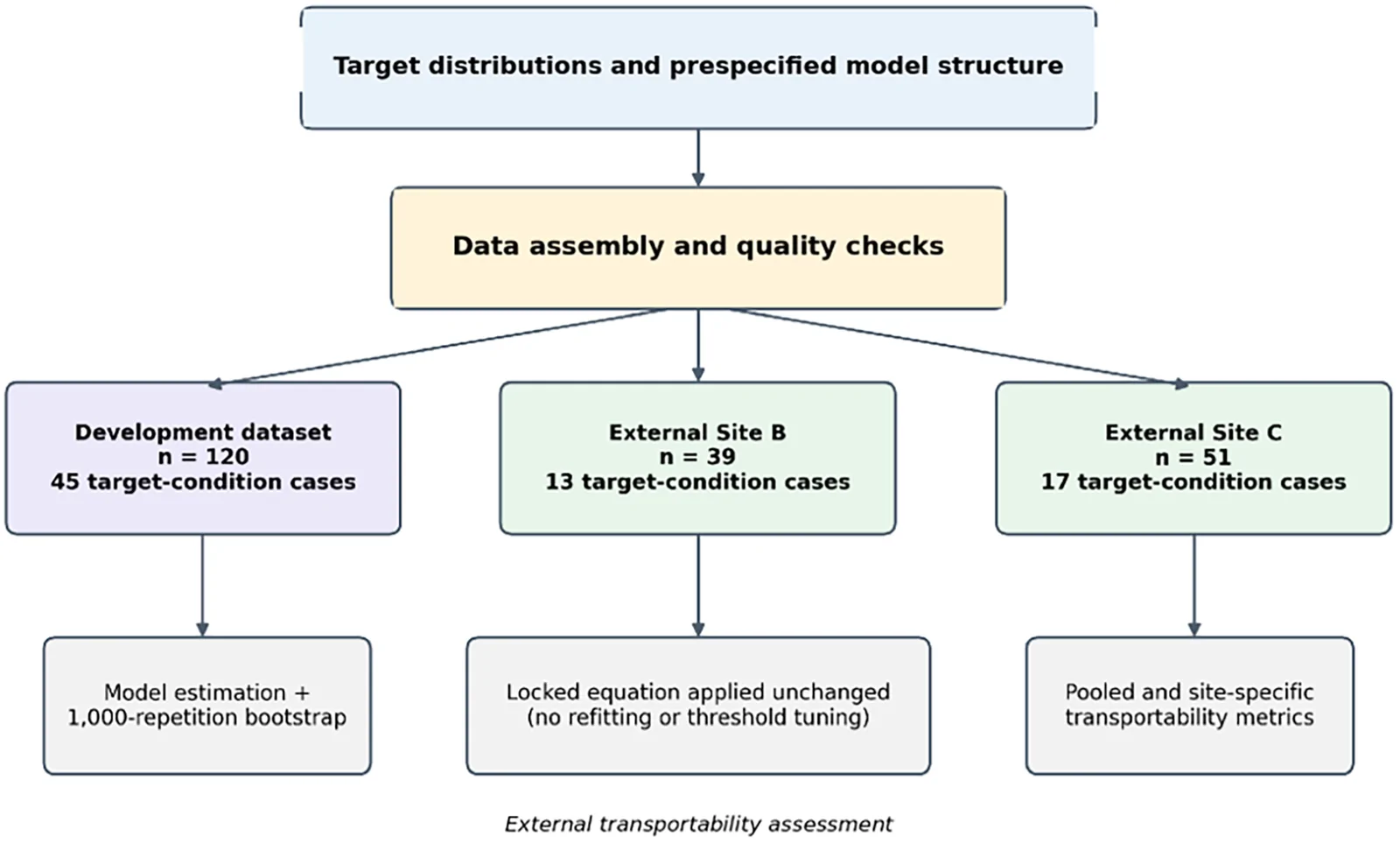
Flow Diagram of development and external validation cohorts.

The development cohort showed a clinically plausible severity gradient: symptom duration and ICCS symptom scores were highest in target-condition records, intermediate in functional-LUTD records, and lowest in control records. Age and sex distributions were similar across groups, indicating that neural, functional, structural, and inflammatory domains primarily represented the diagnostic signal. Cohort characteristics are shown in **Supplementary Tables S6**–**S8**.

### 3.2 Ultrasound, urodynamic, and neuroelectrophysiological profiles

In the development dataset, the target-condition group showed a coherent structural-remodeling profile, with higher detrusor thickness, lower bladder-neck mobility, and higher outlet-discordance markers than the functional-LUTD and control groups. These differences supported the hypothesis that sacral-pathway abnormality can be accompanied by structural and dynamic outlet variability (**Figure 4**, **Table 1**).

**Figure 4:**
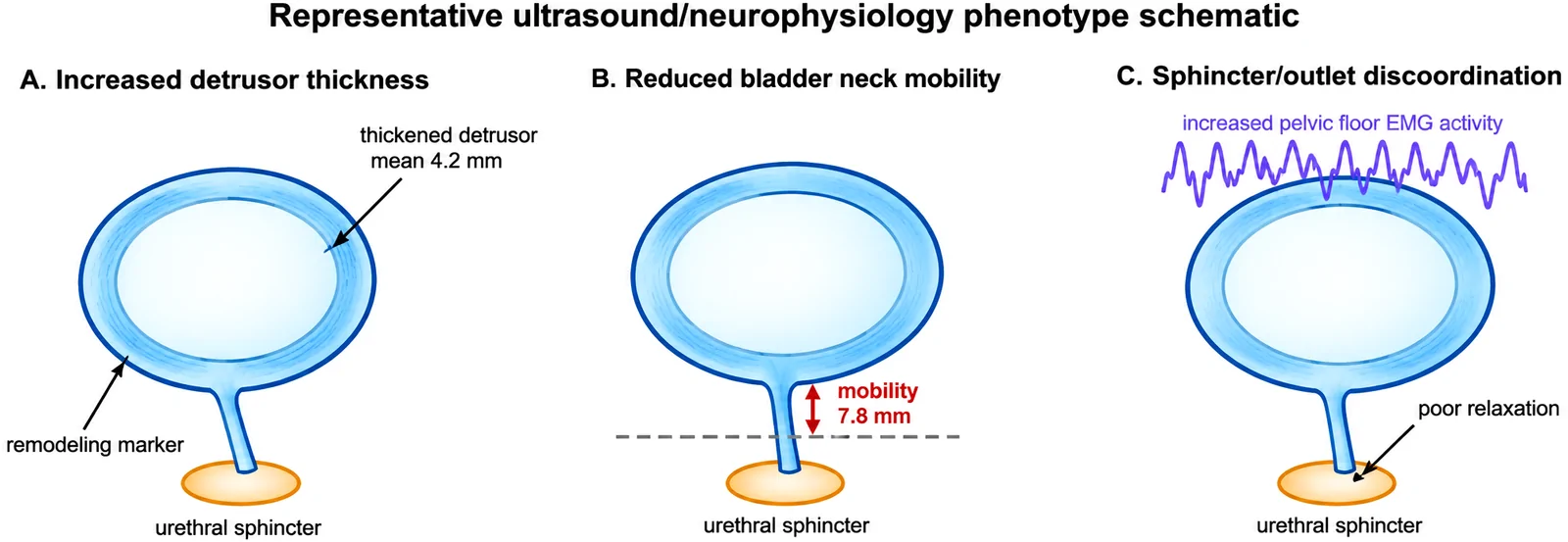
Representative schematic of the high-frequency ultrasound/neurophysiology phenotype, showing increased detrusor thickness, reduced bladder neck mobility, and sphincter/outlet discoordination. **(A)** Increased detrusor thickness, **(B)** Reduced bladder neck mobility, **(C)** Sphincter/outlet discoordination.

**Table 1:** Multimodal structural, urodynamic, and neuroelectrophysiological findings.

Parameter	Occult sacral neuropathy LUTD	Functional non-neurogenic LUTD	Controls	Pairwise p value
Bladder neck mobility, mm	7.8 ± 2.6	10.9 ± 3.1	8.6 ± 2.2	<0.001
Detrusor thickness, mm	4.2 ± 1.1	3.2 ± 0.9	2.1 ± 0.4	<0.001
Urethral sphincter thickness, mm	6.3 ± 1.5	4.9 ± 1.1	4.2 ± 0.8	<0.001
Bladder compliance, mL/cmH2O	9.4 (6.1-13.6)	17.8 (12.5-23.9)	30.4 (24.2-38.8)	<0.001
Detrusor pressure, cmH2O	42.6 ± 13.8	30.8 ± 10.6	18.9 ± 6.2	<0.001
Maximum flow rate, mL/s	7.9 ± 3.1	10.6 ± 3.8	15.2 ± 4.1	<0.001
Post-void residual, mL	38 (18-72)	19 (8-35)	5 (2-10)	<0.001
Sacral reflex latency, ms	45.2 ± 8.7	33.1 ± 6.4	27.6 ± 4.9	<0.001
Abnormal pelvic floor EMG, n (%)	32 (71.1)	20 (44.4)	3 (10.0)	0.010*

*Using Pearson χ^2^ without continuity correction. Fisher’s exact test gives P = 0.018, which leads to the same conclusion. P values represent pairwise comparisons between the occult sacral neuropathy-associated LUTD and functional non-neurogenic LUTD groups. Continuous variables reported as mean ± SD were compared using independent-samples t-tests, variables reported as median (IQR) using the Mann–Whitney U test, and categorical variables using the χ^2^ test. Controls are presented descriptively to provide clinical context and were not included in the reported pairwise P values.

Urodynamic and neuroelectrophysiological distributions further differentiated the groups. Target-condition records were characterized by lower bladder compliance, greater detrusor pressure, lower maximum flow, increased post-void residual, prolonged sacral-reflex latency, and more frequent pelvic-floor EMG abnormalities than comparator records.

### 3.3 Urinary inflammatory and neurotrophic biomarker expression

The urinary inflammatory and neurotrophic markers varied stepwise across the target-condition, functional-LUTD, and control groups. Urinary IL-6, IL-8, TNF-alpha, NGF, and MCP-1 concentrations were highest in the occult sacral neuropathy-related LUTD group, intermediate in the functional non-neurogenic LUTD group, and lowest in controls, consistent with the proposed neuroimmune phenotype (**Table 2**).

**Table 2:** Urinary inflammatory and neurotrophic biomarkers normalized to creatinine.

Biomarker	Occult sacral neuropathy LUTD	Functional non-neurogenic LUTD	Controls	Pairwise p value
IL-6, pg/mg creatinine	8.4 (5.8-12.5)	5.1 (3.4-7.2)	2.2 (1.4-3.0)	<0.001
IL-8, pg/mg creatinine	31.6 (22.4-45.9)	19.4 (12.5-29.6)	8.7 (5.3-12.1)	<0.001
TNF-alpha, pg/mg creatinine	6.9 (4.8-9.4)	4.2 (2.9-6.1)	2.0 (1.2-2.8)	<0.001
NGF, pg/mg creatinine	78.3 (55.7-108.9)	46.8 (31.2-66.5)	18.6 (12.4-27.5)	<0.001
MCP-1, pg/mg creatinine	182 (128-248)	124 (86-176)	62 (38-91)	<0.001

Values are median (IQR). P values represent pairwise comparisons between the occult sacral neuropathy-associated LUTD and functional non-neurogenic LUTD groups using the Mann–Whitney U test. Controls are presented for descriptive reference and were not included in the reported pairwise comparisons.

### 3.4 Diagnostic-model development, internal validation, and external validation (transportability) performance

The exploratory single-domain classifiers had moderate-to-strong development-cohort discrimination (AUC, 0.78-0.86), and the integrated multimodal classifier had an AUC of 0.94 (**Figures 5A**, **B**, **Table 3**). The prespecified locked six-predictor logistic model had an apparent AUC of 0.937, Brier score of 0.099, sensitivity of 82.2, specificity of 88.0, positive predictive value of 80.4, and a negative predictive value of 89.2. Average calibration slope and intercept were 1.171 and -0.058, respectively. There was an AUC optimism of 0.018 across 1,000 bootstrap repetitions, the optimism-corrected AUC of 0.920, and the optimism-corrected Brier score of 0.116. The average intercept of the bootstrap test calibration was -0.065, and the average slope of the calibration was 1.032.

**Figure 5:**
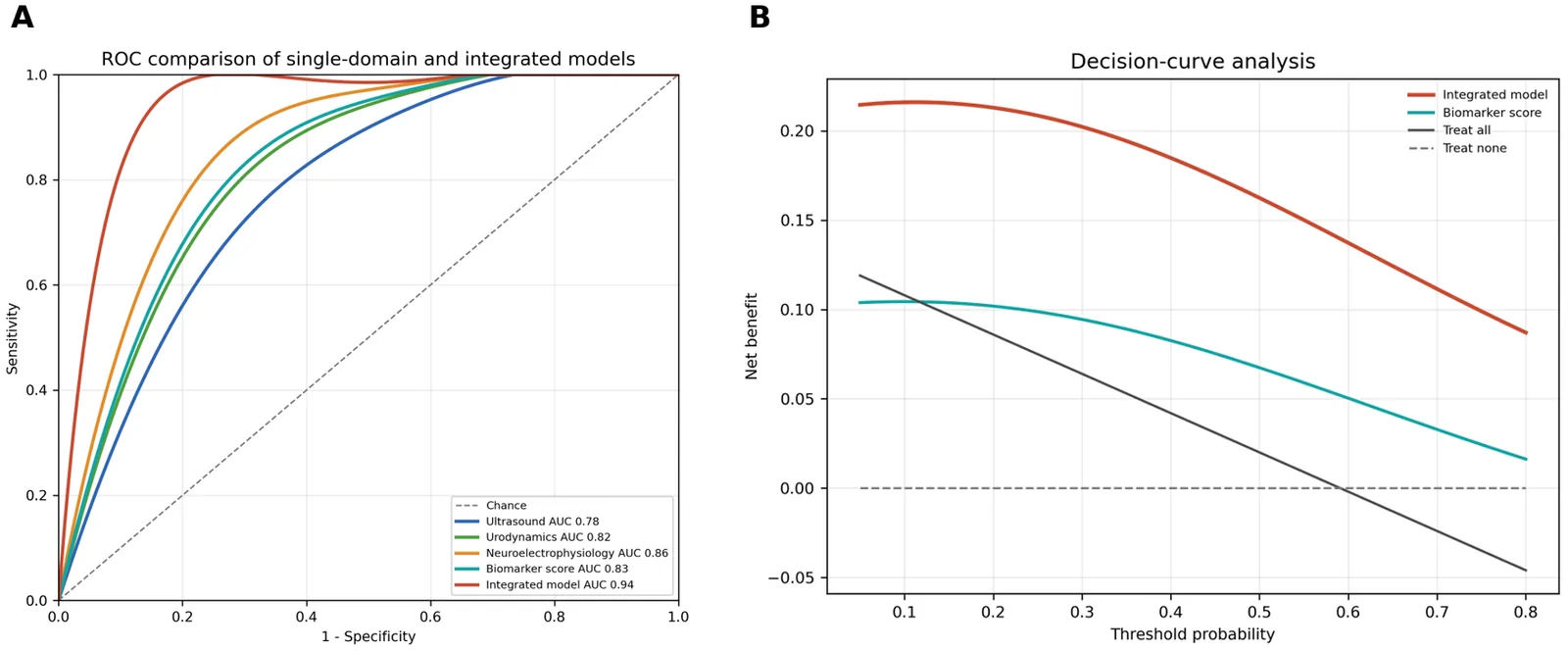
**(A)** ROC comparison of individual diagnostic domains and the integrated multimodal neuroimmune model in the development dataset. **(B)** Decision-curve analysis for the development dataset.

**Figure 6:**
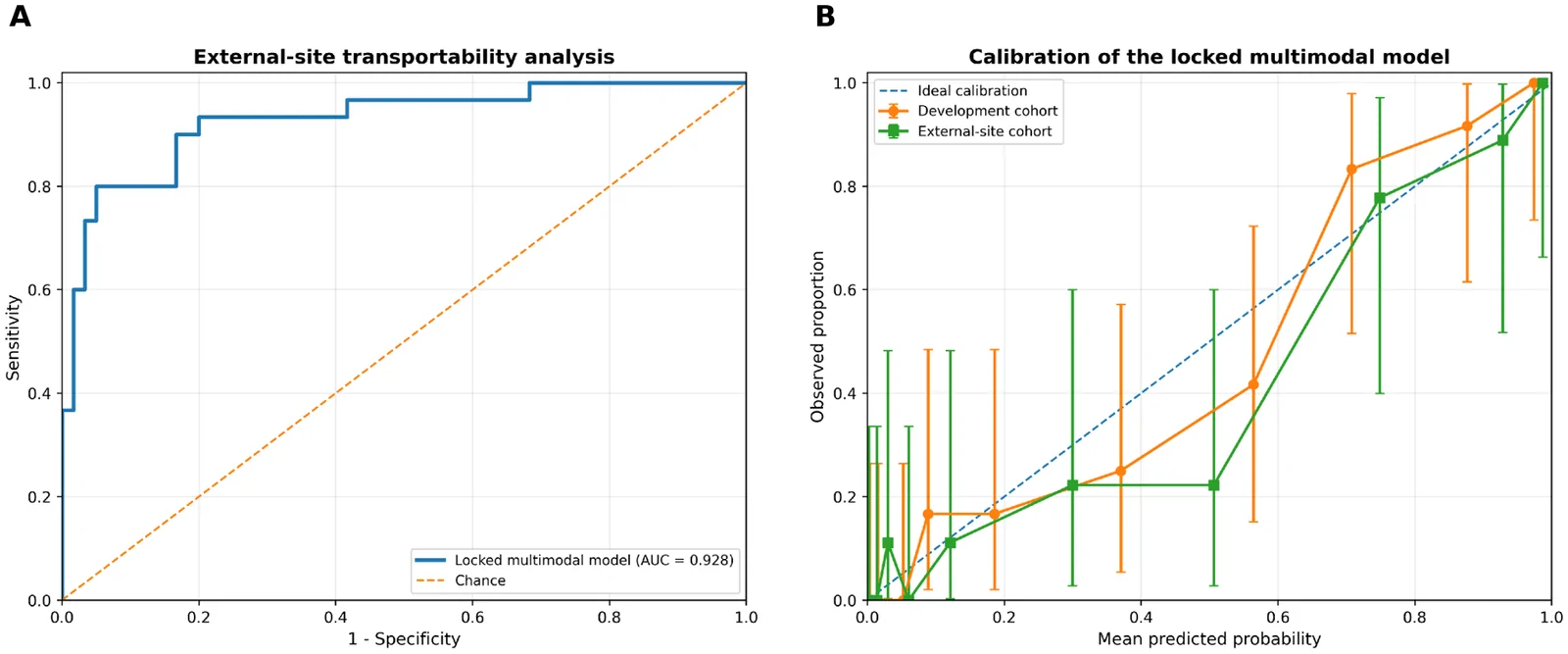
**(A)** Receiver-operating-characteristic curve for the locked model in the external-site transportability dataset. **(B)** Calibration of the locked model in the development and external-site transportability datasets. Points represent probability groups with 95% binomial confidence intervals.

**Table 3:** Exploratory development-cohort diagnostic accuracy of individual domains and the integrated classifier.

Model	AUC (95% CI)	Sensitivity %	Specificity %	PPV %	NPV %	p vs integrated
High-frequency ultrasound	0.78 (0.69-0.86)	71.1	74.7	61.5	81.7	<0.001
Urodynamics	0.82 (0.74-0.89)	77.8	78.7	68.6	85.5	<0.001
Neuroelectrophysiology	0.86 (0.79-0.92)	82.2	84.0	75.5	88.7	0.012
Urinary biomarker score	0.83 (0.75-0.90)	80.0	80.0	70.6	86.9	0.004
Integrated multimodal model	0.94 (0.89-0.98)	88.9	90.7	86.9	92.1	Reference

In the external validation analysis, the AUC was 0.928 (95% CI, 0.861-0.977), whereas the Brier score was 0.098 when the locked equation was applied without refitting to the pooled external-site data. The calibration intercept (-0.400) suggested moderate overprediction in the external sample, and the calibration slope (0.887) indicated mildly over-extreme predictions. Site-based AUCs were 0.923 at Site B and 0.939 at Site C. With the prespecified 0.50 cut-off, pooled sensitivity was 80.0, specificity was 88.3, positive predictive value was 77.4, and negative predictive value was 89.8. **Table 4** and **Figures 6A**, **B** show the external ROC and calibration plots.

**Table 4:** Locked-model development, internal-validation, and external-validation performance.

Cohort/analysis	AUC	Brier	Sensitivity/specificity	PPV/NPV	Calibration intercept	Calibration slope
Development apparent	0.937	0.099	82.2%/88.0%	80.4%/89.2%	-0.058	1.171
Bootstrap optimism-corrected	0.920	0.116	–	–	-0.065	1.032
External pooled (n=90)	0.928	0.098	80.0%/88.3%	77.4%/89.8%	-0.400	0.887
External Site B (n=39)	0.923	0.095	69.2%/92.3%	81.8%/85.7%	0.148	0.903
External Site C(n=51)	0.939	0.100	88.2%/85.3%	75.0%/93.5%	-0.828	0.968

External pooled AUC 95% CI, 0.861-0.977. Bootstrap-corrected calibration values are the mean test sample across 1,000 bootstrap repetitions.

**Figure 7:**
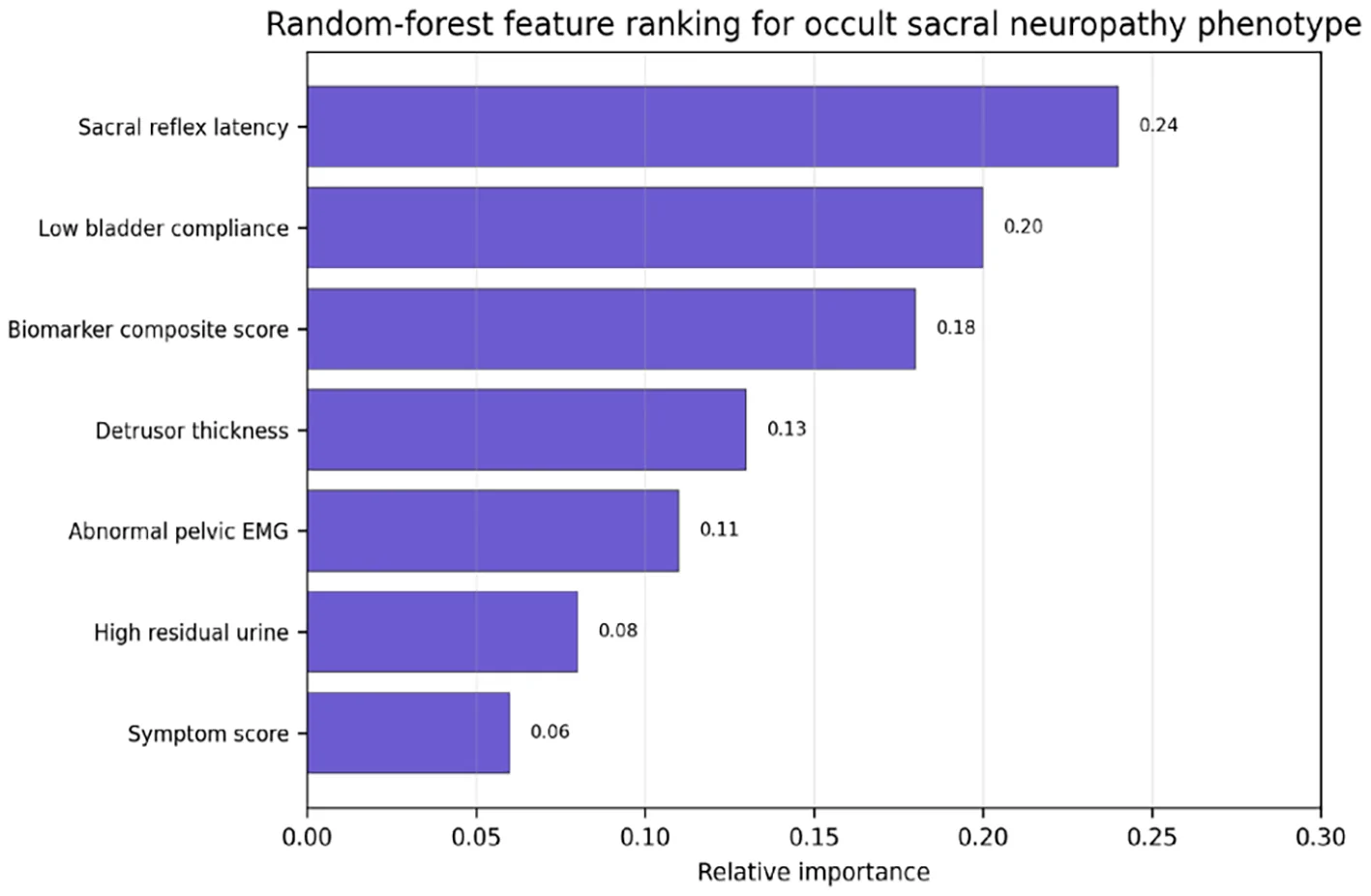
Feature importance ranking from exploratory random-forest classification in the development dataset.

**Figure 8:**
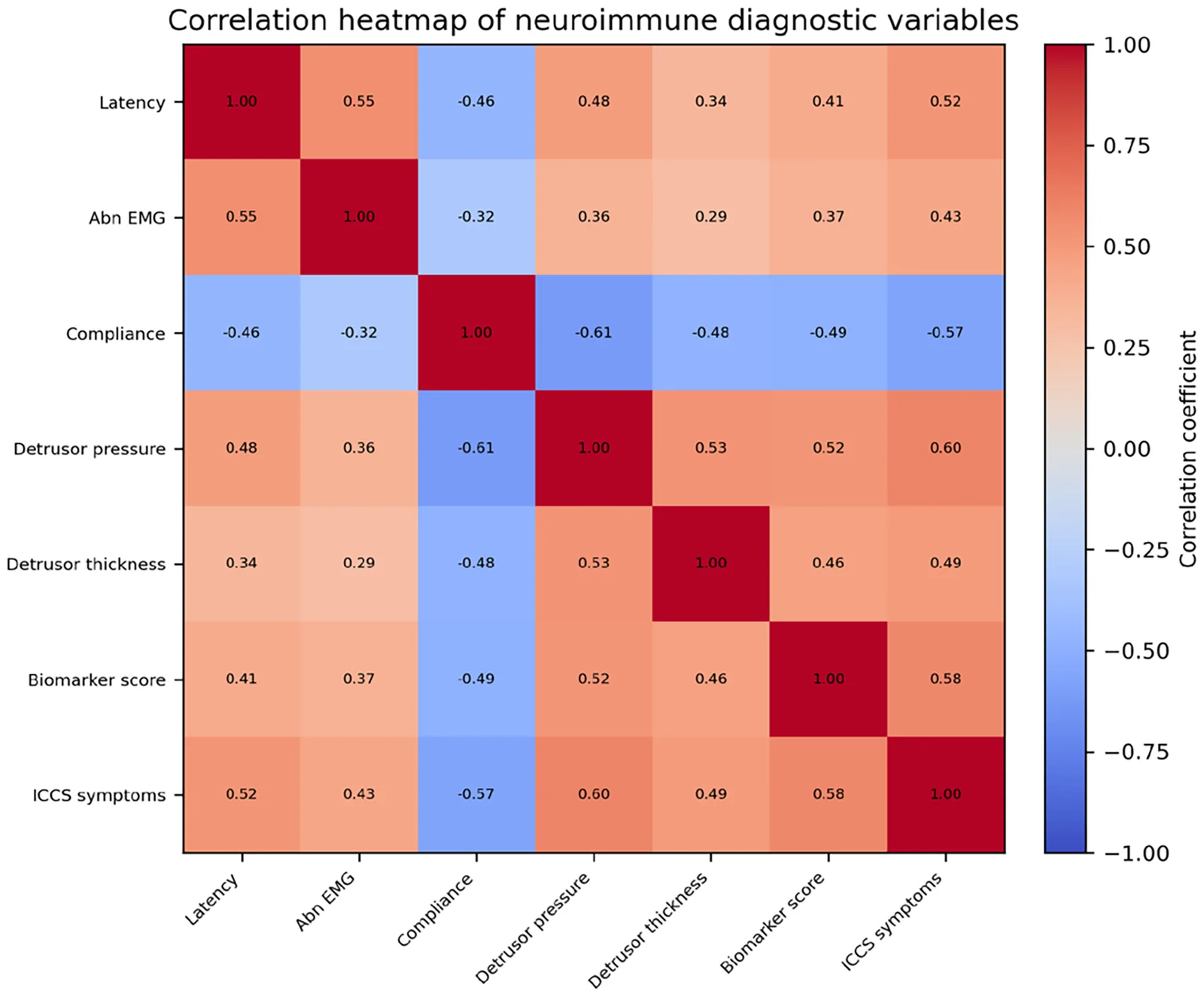
Correlation heatmap of variables in the development dataset. Positive values indicate direct association; negative values indicate inverse association.

### 3.5 Predictors and correlation structure of the neuroimmune phenotype

After controlling for age and sex, the exploratory association model identified prolonged sacral-reflex latency, low bladder compliance, high biomarker composite score, and increased detrusor thickness as independent correlates, which differed conceptually from the locked continuous-predictor diagnostic equation (**Table 5**). Random-forest feature ranking identified the same domains as the most prominent (**Figure 7**). Correlation analysis showed that the biomarker composite was positively associated with ICCS symptom score, detrusor pressure, and detrusor thickness, and negatively associated with bladder compliance, indicating that neural, inflammatory, and remodeling signals converge (**Table 6**, **Figure 8**).

**Table 5:** Multivariable predictors of occult sacral neuropathy-related LUTD.

Predictor	Adjusted OR	95% CI	p value	Interpretation
Sacral reflex latency ≥38 ms	5.42	2.10-13.98	0.001	Strong independent neural predictor
Bladder compliance <12 mL/cmH2O	4.21	1.58-11.24	0.004	Functional remodeling predictor
Elevated biomarker composite score	3.86	1.48-10.05	0.006	Neuroimmune activation predictor
Detrusor thickness ≥3.5 mm	2.71	1.04-7.06	0.041	Structural remodeling predictor
Age, per year	1.03	0.92-1.15	0.62	No independent association
Male sex	1.18	0.49-2.83	0.71	No independent association

These threshold-based estimates were exploratory and were separate from the primary locked diagnostic model, in which continuous predictors were retained and standardized without dichotomization.

**Table 6:** Key correlations supporting the neuroimmune pathway.

Variable pair	Correlation coefficient	p value	Direction of effect
Biomarker composite score vs ICCS symptom score	r = 0.58	<0.001	A higher immune signal is associated with greater symptoms
Biomarker composite score vs detrusor pressure	r = 0.52	<0.001	Higher immune signal associated with pressure elevation
Biomarker composite score vs bladder compliance	r = -0.49	<0.001	A higher immune signal is associated with lower compliance
Sacral reflex latency vs abnormal EMG activity	r = 0.55	<0.001	Delayed reflex associated with outlet discoordination
Detrusor thickness vs bladder compliance	r = -0.48	<0.001	A thicker detrusor is associated with lower compliance

## 4 Discussion

### 4.1 Principal findings

This study characterized a candidate multipolar neuroimmune diagnostic phenotype and evaluated the model using bootstrap internal validation and independent external validation. The locked six-predictor model showed good discrimination after 1,000-repetition bootstrap optimism reduction (AUC, 0.920), with an average calibration intercept near zero and a slope near one. When applied to an independent two-site dataset without refitting, it yielded an AUC of 0.928, a calibration slope of 0.887, and a negative calibration intercept indicating moderate overprediction. These results highlight the need to understand discrimination and calibration together, rather than relying on AUC alone (20, 21).

This aligns with pediatric continence standards, which posit that lower urinary tract symptomatology should be interpreted using child-specific terminology, developmental context, and conventional urodynamic reporting (1, 2, 15, 22–24). It also aligns with more recent pediatric neurogenic bladder recommendations, which base risk stratification on functional bladder behavior, upper-tract protection, and close monitoring rather than symptoms alone (3). The innovation of the current framework is that it adds an immunologically informed component to the existing structural and functional assessment pathway.

### 4.2 Linking neural dysfunction to inflammatory activation

The key biological message is the association between dysfunction of occult sacral pathways and urinary immune activation. Micturition requires coordinated afferent stimulation, autonomic discharge, and relaxation of the detrusor and sphincter muscles (5, 6, 13). The urothelium is not a passive barrier; rather, it is an active interface of the senses and the immunological response that can respond to stretch, neural stimuli, infection-associated stimuli, and inflammatory mediators (7). Thus, the self-reinforcing pathway of chronic sacral dysregulation can plausibly bring about urothelial stress, the release of local cytokines and neurotrophins, afferent sensitization, and a progressively remodeled detrusor (25).

In the latter framework, high IL-6, IL-8, TNF-alpha, NGF, and MCP-1 are not considered disease-specific markers on their own (25, 26). Cytokine elevations can also result from infection, irritation, overdistension, or other inflammatory conditions. They are valuable because they are correlated with delayed sacral reflex latency, abnormal pelvic floor EMG, low bladder compliance, high detrusor pressure, and detrusor thickening. This convergence between findings supports a neuroimmune phenotype. Recent neuroimmune biology also suggests that neuroimmune signaling can link nervous system activity and immune signaling, forming clinical feedback loops that regulate one another (8).

### 4.3 Potential diagnostic and clinical implications

The hypothesis for future clinical testing is that persistent urgency, interrupted stream, straining, escalated residual urine, or frequent urinary symptoms might be examined when structural, urodynamic, neuroelectrophysiological, and inflammatory results converge on three or four points. The current analysis cannot define referral thresholds, surveillance, treatment choice, or the value of neuromodulation. Prospective diagnostic-impact studies in pediatric populations are needed to address those questions (27).

The combined model is still experimental. The external transportability analysis enhanced the methodological assessment by testing a locked equation across shifted site distributions. Therefore, the model cannot be considered a conclusive diagnostic and treatment-selection criterion until it has been tested prospectively across multiple clinical centers using the same definitions of predictors, reference-standard adjudication, and preanalytical biomarker controls (16, 20).

### 4.4 Why multimodal profiling is stronger than single-test interpretation

Single-test interpretation is susceptible to misclassification (13, 14). Urodynamics can define high pressure, low compliance, or poor flow, but not the upstream neural mechanism. Ultrasound may show detrusor thickening and bladder neck mobility, although, again, this could be secondary to chronic symptoms. Depending on the biomarkers, it is possible to demonstrate inflammatory activity but not to determine its type: neural, infectious, irritative, or nonspecific. Neuroelectrophysiology can assess sacral delay or EMG abnormalities, but not tissue remodeling or inflammatory biology. The integrated model is more robust because it asks whether these domains point in the same direction (7–9).

This is especially important in children, since bladder capacity, cooperation during testing, symptom reporting, pelvic floor maturity, and neurodevelopmental growth vary with age. Age-stratified interpretation should therefore be considered when interpreting multimodal diagnostic findings. Different age groups may require age-adjusted cut-off levels rather than a single universal threshold. This consideration is particularly important for sacral reflex latency and urodynamic variables such as bladder capacity, in which developmental physiology can influence normal values (2, 3).

### 4.5 Strengths and limitations

Major strengths included clear data labeling, inclusion of symptomatic comparators, multimodal domain coverage, an explicit composite reference standard, full reporting of locked equations, bootstrap optimism correction, and simultaneous evaluation of discrimination, overall prediction error, and calibration. Reproducibility was also strengthened by explicit preanalytical biomarker procedures.

The development and external datasets were small. The analysis cannot fully support clinical prevalence, spectrum effects, assay variability, missingness, adjudication error, or real-world transportability. Coefficient and calibration uncertainty is also material because the development sample was small relative to the candidate domains. Further patient-level development and geographically diverse external validation are required. Although we performed bootstrap and independent validation, the relatively modest sample size may still limit the precision of coefficient estimates and calibration. The study did not systematically capture detailed prior bladder-directed medication exposure; therefore, it cannot rule out potential treatment-related effects on symptoms and functional bladder measurements.

## 5 Conclusion

The multimodal neuroimmune model linked sacral neural dysfunction, non-optimal bladder mechanics, structural remodeling, and urinary inflammatory activity. The fixed equation showed discrimination after bootstrap optimism adjustment and exhibited mild miscalibration under a two-site distribution shift. The clearly defined reference standard, full equation, calibration intercept and slope, and stated biomarker-handling procedures enhance reproducibility. Before clinical application, prospective multicenter external validation and clinical-impact assessment are required.

## Data Availability

The original contributions presented in the study are included in the article/**Supplementary Material**. Further inquiries can be directed to the corresponding author.

## Supplementary material

Supplementary Table 1 Scientific rationale for multimodal neuroimmune diagnostic profiling.

Supplementary Table 2 Operational components of the composite reference standard.

Supplementary Table 3 Urinary biomarker preanalytical and analytical procedures.

Supplementary Table 4 Locked six-predictor model specification.

Supplementary Table 5 Development and external-validation cohort structure and data sources.

Supplementary Table 6 Baseline characteristics of the analytic cohort.

Supplementary Table 7 Baseline characteristics of the external cohort.

Supplementary Table 8 Clinical characteristics of the development and external validation cohorts.
