# Supplementary material for: Multimodal neuroimmune diagnostic profiling for pediatric lower urinary tract dysfunction associated with occult sacral nerve abnormalities
Source: Front Immunol. 2026 Oct 6;17:1947984. doi: 10.3389/fimmu.2026.1947984 (PMC13680195; doi:10.3389/fimmu.2026.1947984)
Supplement: Supplementary file 1 [file Table1.docx]

**Multimodal Neuroimmune Diagnostic Profiling for Pediatric Lower Urinary Tract Dysfunction Associated with Occult Sacral Nerve Abnormalities**

*A Diagnostic Model Development and Validation Study*

**Supplementary Tables**

**Table S1. Scientific rationale for multimodal neuroimmune diagnostic profiling**

| **Diagnostic domain** | **Primary information gained** | **Reason for integration** |
| --- | --- | --- |
| High-frequency ultrasound | Bladder neck mobility, detrusor morphology, sphincter thickness | Identifies structural remodeling that may not be visible in symptoms alone |
| Urodynamics | Bladder compliance, detrusor pressure, flow pattern, residual urine | Defines functional bladder and outlet behavior under filling and voiding conditions |
| Neuroelectrophysiology | Pelvic floor EMG activity and sacral reflex latency | Supports detection of sacral pathway dysfunction and sphincter discoordination |
| Urinary biomarkers | IL-6, IL-8, TNF-alpha, NGF, MCP-1 | Captures inflammatory and neurotrophic activity linked to neuroimmune remodeling |

**Table S2: Operational components of the composite reference standard**

| **Component** | **Operational evidence reviewed** | **Role in final adjudication** |
| --- | --- | --- |
| Clinical phenotype | Persistent or recurrent storage/voiding symptoms, elevated residual urine, recurrent urinary symptoms, or failure of standard functional-LUTD management. | Required clinical context; not sufficient alone. |
| Neurological examination | Documented sacral, perineal, anal-tone/reflex, sensory, gait, or lower-limb findings when present. | Objective supportive evidence. |
| Spinal/pelvic MRI | Low or abnormal conus, thickened/fatty filum, sacral abnormality, or another subtle tethering-related feature; normal or equivocal MRI did not automatically exclude occult dysfunction. | Supportive, not mandatory. |
| Conventional urodynamics | Reproducible low compliance, high-pressure filling/voiding, detrusor-sphincter dyssynergia, sphincter non-relaxation, or another neurogenic pattern. | Supportive; no single threshold was determinative. |
| Longitudinal course | Persistence, progression, recurrence, or specialist neuro-urological assessment across follow-up. | Used to confirm clinical coherence and exclude transient dysfunction. |
| Excluded from label generation | Urinary biomarkers, biomarker composite, and locked-model probability were not used to assign the target-condition label. | Prevented direct incorporation of the model output into its target. |

**Table S3. Urinary biomarker preanalytical and analytical procedures**

| **Stage** | **Procedure** | **Quality-control purpose** |
| --- | --- | --- |
| Collection | Collection before urodynamic catheterization. | Designed to avoid catheter-related inflammatory signals. |
| Clarification | Centrifugation at 3000 or 5000 x g for 10 minutes. | Designed to remove cells and debris while preserving soluble analytes. |
| Aliquoting | 1.0-1.5 mL aliquots. | Designed to reduce repeated freeze-thaw exposure. |
| Storage | Continuous storage at -80 °C; duration encoded for each record. | Represented long-term protein-stability conditions. |
| Freeze-thaw | Maximum two encoded cycles; most development records had zero prior cycles. | Represented limited degradation and cycle-related bias. |
| Assay | ELISA with blinding, standard curve, control, duplicate-well CV, and batch review. | Represented plate validity and reproducibility controls. |
| Normalization | All analytes expressed per urinary creatinine. | Reduced variation from urine dilution. |

**Table S4. Locked six-predictor model specification**

| **Term** | **Development center** | **Development scale** | **Coefficient** |
| --- | --- | --- | --- |
| Intercept | - | - | -1.034813 |
| Sacral-reflex latency, ms | 36.262 | 10.052 | 1.497302 |
| Bladder compliance, mL/cmH2O | 19.659 | 12.053 | -0.738997 |
| Biomarker composite | 0.000 | 0.841 | 0.524326 |
| Detrusor thickness, mm | 3.303 | 1.197 | 0.423860 |
| Age, years | 8.105 | 3.801 | 0.085344 |
| Male sex (1=yes, 0=no) | 0.550 | 0.497 | 0.094346 |

*Model equation: logit(p) = -1.034813 + sum [coefficient x ((predictor - center)/scale)]. The 0.50 classification threshold was fixed before external application.*

**Table S5. Development and external-validation cohort structure and data sources**

| **Group** | **Definition** | **Sample size** | **Core data source** |
| --- | --- | --- | --- |
| Development: target condition | Occult sacral neuropathy-related LUTD records | 45 | Multimodal variables and reference-standard components |
| Development: functional LUTD | Functional non-neurogenic LUTD records | 45 | Multimodal variables |
| Development: controls | Mild/transient urinary-symptom records | 30 | Screening and biomarker variables |
| External cohort | External-site records | 90 | Same locked predictors; shifted distributions; no model refitting |

**Table S6. Baseline characteristics of the analytic cohort**

| **Variable** | **Occult sacral neuropathy LUTD (n=45)** | **Functional non-neurogenic LUTD (n=45)** | **Controls (n=30)** | **Pairwise p** |
| --- | --- | --- | --- | --- |
| Age, years, mean ± SD | 8.2 ± 4.1 | 7.9 ± 3.8 | 8.1 ± 4.0 | 0.723 |
| Male sex, n (%) | 26 (57.8) | 24 (53.3) | 16 (53.3) | 0.832 |
| Symptom duration, months, median (IQR) | 17.6 (10.2-25.8) | 14.9 (8.3-21.5) | 2.8 (1.6-4.2) | 0.305 |
| ICCS urinary symptom score, mean ± SD | 18.2 ± 5.4 | 13.1 ± 4.6 | 3.1 ± 1.5 | <0.001 |
| Daytime incontinence, n (%) | 34 (75.6) | 29 (64.4) | 4 (13.3) | 0.358 |
| Interrupted stream/dribbling, n (%) | 31 (68.9) | 24 (53.3) | 2 (6.7) | 0.194 |
| Post-void residual elevation, n (%) | 28 (62.2) | 17 (37.8) | 1 (3.3) | 0.034 |

*P values represent pairwise comparisons between the occult sacral neuropathy-associated LUTD and functional non-neurogenic LUTD groups; controls are presented descriptively and were not included in these pairwise tests. Continuous variables reported as mean ± SD were compared using independent-samples t-tests, symptom duration using the Mann–Whitney U test, and categorical variables using exact tests, as appropriate.*

**Table S7. Baseline characteristics of the external cohort**

| **Variable** | **Occult sacral neuropathy LUTD (n=30)** | **Functional non-neurogenic LUTD (n=30)** | **Controls (n=30)** | **Pairwise p** |
| --- | --- | --- | --- | --- |
| Age, years, mean ± SD | 8.7 ± 3.8 | 8.1 ± 4.0 | 8.3 ± 4.2 | 0.538 |
| Male sex, n (%) | 17 (56.7) | 16 (53.3) | 16 (53.3) | 1.000 |
| Symptom duration, months, median (IQR) | 19.7 (8.8–30.8) | 17.1 (9.8–26.2) | 3.0 (1.7–4.2) | 0.433 |
| ICCS urinary symptom score, mean ± SD | 17.5 ± 5.9 | 12.6 ± 4.9 | 3.2 ± 1.6 | <0.001 |
| Daytime incontinence, n (%) | 23 (76.7) | 19 (63.3) | 4 (13.3) | 0.399 |
| Interrupted stream/dribbling, n (%) | 21 (70.0) | 16 (53.3) | 2 (6.7) | 0.288 |
| Post-void residual elevation, n (%) | 19 (63.3) | 11 (36.7) | 1 (3.3) | 0.070 |

*P values represent pairwise comparisons between the occult sacral neuropathy-associated LUTD and functional non-neurogenic LUTD groups; controls are presented descriptively and were not included in these pairwise tests. Continuous variables reported as mean ± SD were compared using independent-samples t-tests, symptom duration using the Mann–Whitney U test, and categorical variables using exact tests, as appropriate.*

**Table S8. Clinical characteristics of the development and external validation cohorts**

| **Characteristic** | **Development cohort (n=120)** | **External validation cohort (n=90)** | **p value** |
| --- | --- | --- | --- |
| Occult sacral neuropathy LUTD, n (%) | 45 (37.5) | 30 (33.3) | 0.533 |
| Functional non-neurogenic LUTD, n (%) | 45 (37.5) | 30 (33.3) |  |
| Controls, n (%) | 30 (25.0) | 30 (33.3) |  |
| Age, years (mean) | 8.10 | 8.37 | 0.60* |
| Male sex, n (%) | 66 (55.0) | 49 (54.4) | 0.93 |
| ICCS symptom score (mean) | 12.66 | 11.11 | 0.11* |
| Detrusor thickness (mean, mm) | 3.36 | 3.30 | 0.74* |
| Bladder compliance (median, mL/cmH₂O) | 17.65 | 16.26 | 0.38† |
| Sacral reflex latency (mean, ms) | 35.30 | 36.24 | 0.42* |
| NGF (median, pg/mg creatinine) | 50.93 | 46.34 | 0.35† |
| Abnormal pelvic floor EMG, n (%) | 55 (45.8) | 37 (41.1) | 0.49 |
